# New Insights into Butyrylcholinesterase Activity Assay: Serum Dilution Factor as a Crucial Parameter

**DOI:** 10.1371/journal.pone.0139480

**Published:** 2015-10-07

**Authors:** Joanna Jońca, Monika Żuk, Bartosz Wasąg, Anna Janaszak-Jasiecka, Krzysztof Lewandowski, Bartosz Wielgomas, Krzysztof Waleron, Jacek Jasiecki

**Affiliations:** Medical University of Gdańsk, Gdańsk, Poland; Penn State College of Medicine, UNITED STATES

## Abstract

Butyrylcholinesterase (BChE) activity assay and inhibitor phenotyping can help to identify patients at risk of prolonged paralysis following the administration of neuromuscular blocking agents. The assay plays an important role in clinical chemistry as a good diagnostic marker for intoxication with pesticides and nerve agents. Furthermore, the assay is also commonly used for in vitro characterization of cholinesterases, their toxins and drugs. There is still lack of standardized procedure for measurement of BChE activity and many laboratories use different substrates at various concentrations. The purpose of this study was to validate the BChE activity assay to determine the best dilution of human serum and the most optimal concentration of substrates and inhibitors. Serum BChE activity was measured using modified Ellman’s method applicable for a microplate reader. We present our experience and new insights into the protocol for high-throughput routine assays of human plasma cholinesterase activities adapted to a microplate reader. During our routine assays used for the determination of BChE activity, we have observed that serum dilution factor influences the results obtained. We show that a 400-fold dilution of serum and 5mM S-butyrylthiocholine iodide can be successfully used for the accurate measurement of BChE activity in human serum. We also discuss usage of various concentrations of dibucaine and fluoride in BChE phenotyping. This study indicates that some factors of such a multicomponent clinical material like serum can influence kinetic parameters of the BChE. The observed inhibitory effect is dependent on serum dilution factor used in the assay.

## Introduction

Butyrylcholinesterase (EC 3.1.1.8; BChE), also known as plasma cholinesterase or pseudocholinesterase, is a serine hydrolase present in almost all mammalian tissues with the highest levels detected in plasma and liver [1, 2]. BChE hydrolyzes chemicals containing ester bonds such as: drugs acting at the neuromuscular junction, local anesthetics like: succinylcholine, mivacurium, procaine [3]. The exact physiological function of BChE remains elusive, although it acts as an endogenous bioscavenger for anticholinesterase agents. BChE in the plasma serves as the first line of defense against toxic compounds reaching the bloodstream, that might inhibit acetylcholinesterase activity (AChE; EC 3.1.1.7), a sister enzyme essential for functioning of the nervous system. BChE as well as a pool of AChE localized on the red blood cell surface hydrolyze or sequester the toxic compounds before they reach their targets–synaptic AChE, and therefore provide protection against administrated or inhaled poisons [4]. There is ten times more BChE than AChE in the human body, BChE represents 0.1% of human plasma proteins with its concentration of 2–5 mg/L [5, 6]. Organophosphate (OP) compounds used as pesticides and chemical warfare agents (eg. sarin, VX) are among the most lethal chemicals, due to irreversible inhibition of AChE. OP poisoning is a major public health concern. According to the World Health Organization 200,000 people die annually in developing countries as a result of poisoning by OP pesticides [7]. Exposure to (OP) pesticide or nerve agent is commonly assessed by measuring the decrease in AChE or BChE activities in human blood because these agents inhibit both cholinesterases [8]. The primary toxic effect of OP is inhibition of AChE, however many compounds have a more pronounced toxic effect on BChE than AChE. It has been observed that individuals subjected to pesticide exposure may have reduced BChE activity without clinical signs of poisoning, which indicates that toxins had been sequestered [9]. Since mainly BChE contributes to ChE activity in plasma, therefore it is a very good indicator of OP poisoning, detection of pesticides and nerve agents exposure. However, both AChE and BChE can be measured for monitoring of OP pesticides overexposure [10]. Nevertheless, in clinical toxicology, BChE plasma assay is the most commonly used and preferred method for monitoring of OP intoxicated patients since it is simpler and more reproducible.

Apart from the enzyme activity, DN (dibucaine number) and FN (fluoride number) values are also used in biochemical characterization of BChE patients phenotypes. DN is the percent of BChE activity that is inhibited by dibucaine. DN is used to differentiate individuals who have substitution mutations of the anionic site of the BChE and who are resistant to dibucaine inhibition [11, 12]. The DN and the BChE enzyme activity results can help to identify subjects at risk for prolonged paralysis following the administration of succinylcholine or mivacurium. Decreased BChE enzyme activity in conjunction with a DN less than 30 suggests high risk for prolonged paralysis.

The fluoride variant of BChE owes its name to the observation that it is resistant to inhibition by 0.050 mM sodium fluoride in the in vitro assay. Individuals who are compound heterozygous for the fluoride and atypical alleles (AF phenotype) have a moderately prolonged response and experience about 30 min episodes of apnea after receiving succinyldicholine [13, 14]. The DN, FN and total BChE activity are used in combination to assign a biochemical phenotype [15].

Determination of BChE activity in serum is of interest in various fields of clinical chemistry. A number of studies have shown an association between high serum BChE activity and obesity [16, 17], as well as with insulin resistance and the metabolic syndrome [18, 19], hyperlipidemia [20], coronary artery disease and hypertension [21] and the arterial pathology of diabetes mellitus [22]. BChE has been shown to inactivate ghrelin [23, 24]. However, a recent study using genome-wide analysis suggested that BChE is a marker of the above-mentioned diseases, rather than their cause [25, 26].

A variety of methods have been developed for the determination of ChE activity [27], however the most common assay is based on Ellman’s colorimetric method developed in 1961 and universally used until now because it is rapid, simple, cheap and can be easily adapted for high-throughput analysis [28, 29, 30, 31].

## Materials and Methods

S-butyrylthiocholine iodide (BTC, cat. no. 20820), propionylthiocholine iodide (PTC, cat. no. P2880), 5,5'-dithiobis(2-nitrobenzoic acid) (DTNB, Ellman’s reagent cat. no. D8130), dibucaine hydrochloride (cat. no. D0638), sodium fluoride (cat. no. 201154) were purchased from Sigma-Aldrich (Germany). 0.1 M sodium phosphate buffer (PB, pH 7.4) was prepared by adding 3.1 g of NaH_2_PO_4_•H_2_O and 10.9 g of Na_2_HPO_4_ (anhydrous) to distilled H_2_O (final volume 1 L), and was sterilized. Stock solutions of BTC (100mM in water), PTC (100mM in water), DTNB (10mM in PB) were stored in aliquots at – 20°C and after thawing were used only once. 2mM DTNB, 10 mM BTC or PTC solutions were prepared directly prior to addition to the samples in 100 mM PB, pH 7.4. Stock solutions of dibucaine (10mM) were prepared by dissolution of 3.78 mg dibucaine hydrochloride in 1 ml of distilled water. 0.25 mM dibucaine solution ready to add to reaction mixture was prepared in 100 mM PB, pH 7.4. Stock solutions (20mM) of sodium fluoride were prepared by dissolution of 8.4 mg sodium fluoride in 10 ml of distilled water. 1 mM fluoride solution ready to add to reaction mixture was prepared in 100 mM PB, pH 7.4. All solutions used in assays were prewarmed to 25°C prior to use, unless indicated otherwise.

### Sample preparation

Human blood and serum were procured from a collection of the Central Clinical Laboratory of the Medical University of Gdańsk. The Ethical Committee of the Medical University of Gdańsk waived the need for consent from donors. The study was approved by the local Ethical Committee of Medical University of Gdańsk (NKBBN/304/2013). Serum samples were stored in 0.3 ml aliquots at – 80°C. Prior to analysis 10 μl of serum was added to 190 μl of 100 mM PB pH 7.4 to achieve the first 20-fold dilution, and appropriate further dilutions were prepared as described below. The dilutions of serum were prepared in a microtiter plate, which is convenient in case of high number of samples. It is very important to mix dilutions thoroughly after addition of serum, since serum density and viscosity cause sedimentation of sample to the bottom of the wells. Our experience showed that mixing by pipetting up and down 5 times is sufficient to obtain good suspensions of serum in a volume of 200 μl. Genotypes of patients with UU (**U**sual/**U**sual), UA (**U**sual/ **A**typical) and UF (**U**sual/**F**luoride Resistant) phenotypes were established by direct sequencing of the *BChE* coding sequence. Genotype of atypical heterozygous patient was NM_000055.2:c.293A>G/p.D98G, whereas UF individual (Fluoride–2 variant) was NM_000055.2:c.1253G>T/p.G418V (residues number according to NCBI database).

### Micro-Ellman assay for measuring BChE activity

Butyrylcholinesterase activity was determined spectrophotometrically by modified Ellman's method using BTC or PTC as a substrate [32]. The assay was performed in 96-well microtiter plates in a final reaction volume of 200 μl of 100mM PB buffer (pH 7.4) with a final concentration of 0.5 mM DTNB and 5 mM BTC or PTC (or in different concentrations if required in some experiments). Briefly, 10 μl of diluted serum samples were added to the wells of a microtiter plate containing 40 μl of 100mM PB buffer (pH 7.4), then 50 μl of DTNB (2mM in PB) was added, and incubated for 10 min at 25°C in a microplate-reader to achieve temperature equilibrium and complete the reaction of sample serum proteins' sulfhydryl groups with DTNB. At this step, if needed inhibitors were also added at desired concentrations. The reactions were initiated by addition of 100 μl BTC or 100 μl PTC (10mM in PB). Prior to start of read-out, the microplate was shaken orbitally for 30s with 1 milimeter amplitude at frequency 87.6 rpm in a microplate reader. The absorbance was monitored at 412 nm by repeated measurements at 1 min intervals for at least 5 minutes by a thermostated microplate reader spectrophotometer (Tecan Infinite M200Pro) at 25°C. Calculated enzyme activity was corrected for spontaneous hydrolysis of the substrate and DTNB degradation, for this purpose the wells containing all ingredients except serum were read as blanks. All experiments were repeated three times and the measurements and calculations were conducted with Tecan Magellan V7.0 software.

## Results

### Optimal dilution of serum for BChE activity assay

**Fig 1:**
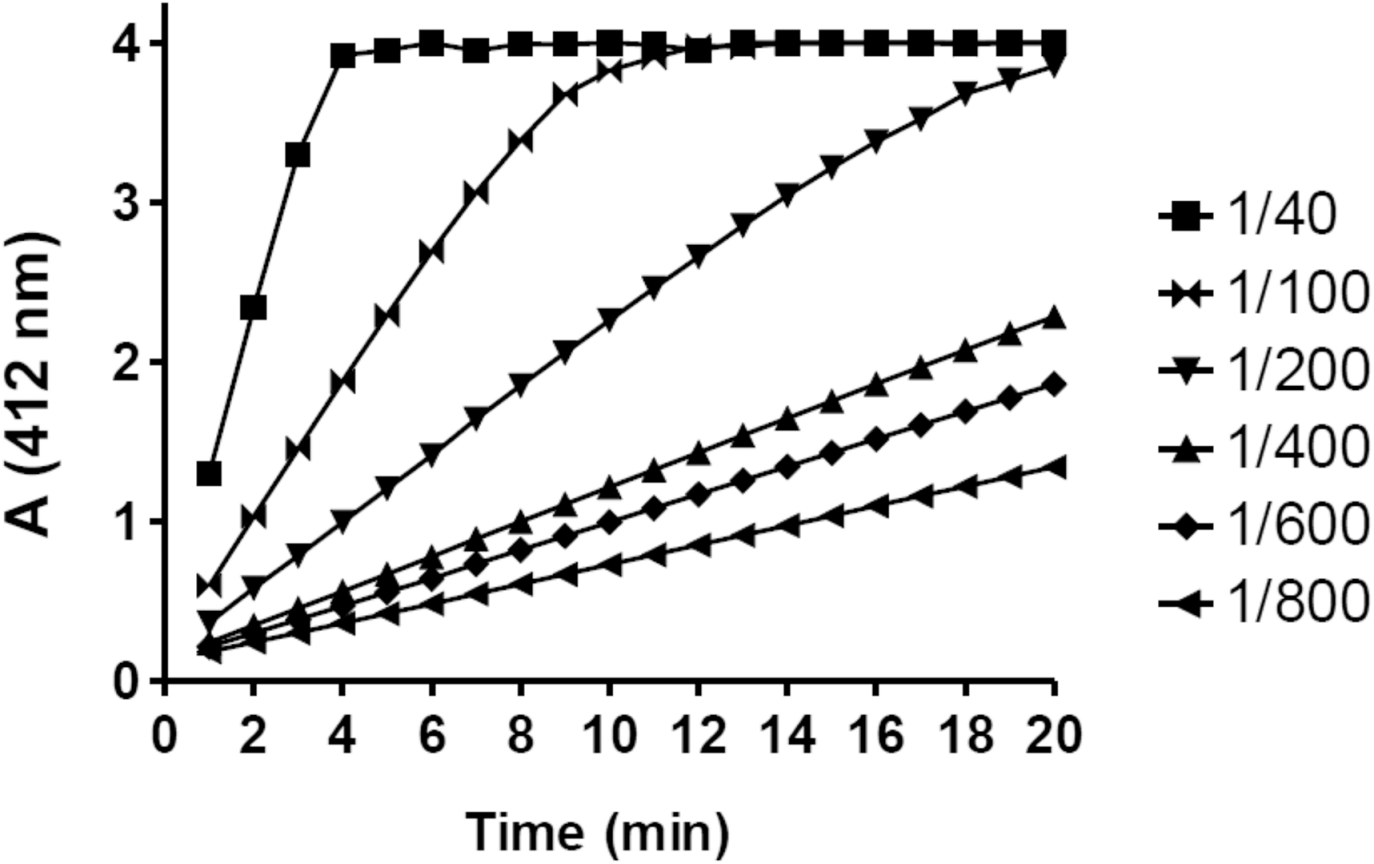
Optimization of the BChE assay linearity. Measurement of TNB ion concentration at 412 nm produced in Ellman's reaction in the presence of 5 mM BTC and 40 (5), 100 (2), 200 (1), 400 (0.5), 600 (0.3), 800 (0.25)-fold diluted serum. Volume (μl) of serum in the reaction mixture (200 μl total volume) is shown above in brackets. Assays were performed in triplicate, standard error for all points is less than 2.5% of the values.

The optimal serum dilution for the BChE activity assay was determined by measuring the absorbance of TNB ions at 412 nm (A412) in 40, 100, 200, 400, 600, 800-fold diluted serum in Ellman reactions. The absorbance was read every 1 minute for 20 minutes at 25°C. A linear relationship between absorbance and BTC concentration was observed across at least 5 points for all samples that were diluted 100-fold or more (Fig 1), but nearly a perfect R^2^—coefficient of determination >0.995 was observed in samples that were diluted 400-fold or more. The minimal serum dilution that shows nearly ideal linear slope for at least 10 minutes was 1/400.

### Determination of appropriate substrate concentration in the assay

**Fig 2:**
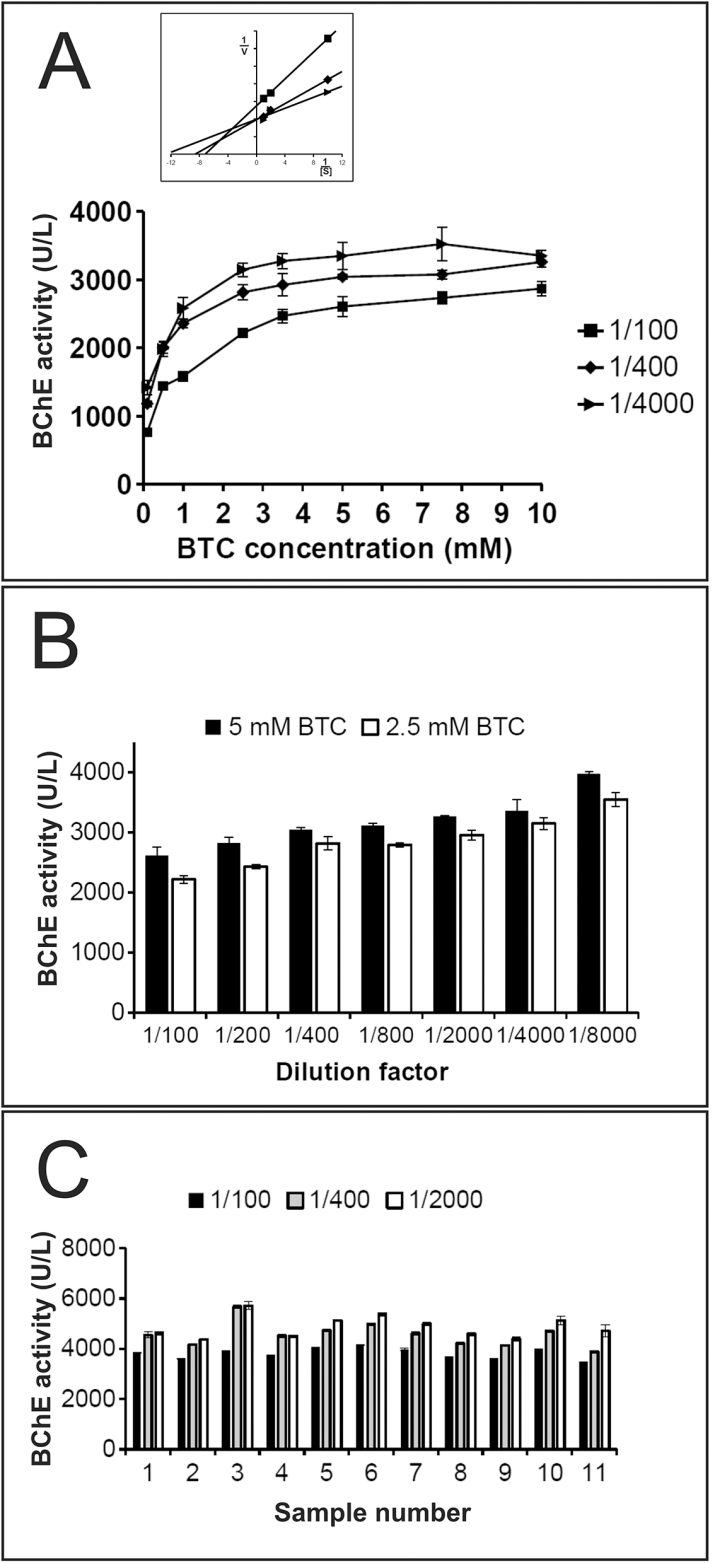
The effect of serum dilution on BChE activity. The activity was estimated based on the mean of hydrolysis rate (OD/min) determined from at least 5 spectrophotometer reads (every 1 minute) to ensure linearity and regularity of slopes. Assays were done in triplicate, error bars represent mean ± standard error (Student’s t-test, p<0.05). **A.** The effect of BTC concentration on the BChE activity in 100, 400, 4000 -fold diluted serum samples. Inset shows a Lineweaver–Burk replot of the results presented in Fig 2A at [BTCh] = 0.1, 0.5 and 1 mM. **B.** The effect of 5mM and 2.5 mM BTC concentration on the BChE activity in variously diluted serum samples. **C.** BChE activity in 100, 400, 2000 -fold diluted eleven randomly selected serum samples from our serum collection samples.

**Fig 3:**
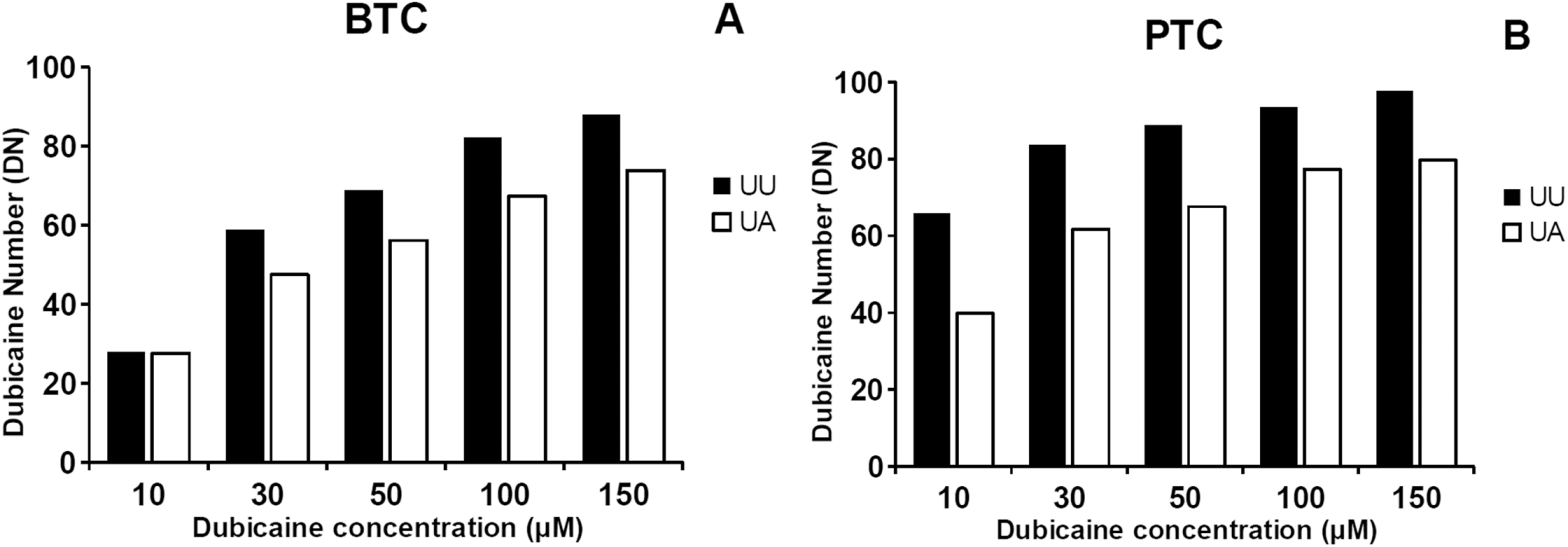
Characteristics of dibucaine inhibition of “usual” and “atypical” forms of BChE. DN values were calculated in assays performed at various dibucaine concentration at 25°C using 5mM BTC (A) or PTC (B) as substrate with two types of 400-fold diluted serum samples from heterozygous individuals with usual UU and UA phenotype. Dibucaine Number (DN) = (1 - (butyrylcholinesterase activity in presence of inhibitor/butyrylcholinesterase activity in absence of inhibitor)) x 100. Assays were performed in triplicate, standard error for all points is less than 3% of the values.

Appropriate BTC concentration in the BChE activity assay was determined in variously diluted serum samples, in the presence of 0.1; 0.5; 1; 2.5; 3.5; 5; 7.5 and 10 mM BTC. The effect of the substrate concentration on the BChE activity in variously diluted serum samples is shown in Fig 2. Concentration of 5 mM BTC at 400-fold diluted serum seems to be optimal and was chosen for the subsequent investigations. It is noteworthy that observed BChE activity in the same serum sample after recalculations was different in various serum dilutions. The more diluted serum, the higher activity of BChE was measured. For the clarity on the Fig 2A only 100, 400, 4000 -fold diluted serum samples were shown. More examples, 100, 200, 400, 800, 2000, 4000, 8000 -fold diluted serum samples at 2.5 mM and 5 mM BTC are presented in Fig 2B. The same effect was observed at other BTC or PTC concentrations (data not shown). Dependence of the serum dilution factor on BChE activity was observed in many samples (Fig 3C).

### Selection of optimal dibucaine concentration

It has been demonstrated that the level of inhibition of BChE by dibucaine depends on its concentration and a type of a substrate [32]. To determine the optimal dibucaine concentration for measurement of dibucaine number (DN), BChE activity was assayed using various dibucaine concentrations at 25°C using 5mM BTC or PTC as substrates. The assays were performed with two types of 400-fold diluted serum samples: from a patient with usual UU phenotype and from a heterozygous patient with UA phenotype. Values of DN >80 for UU phenotype are achieved at least at 100 μM concentration of dibucaine for BTC and 30 μM dibucaine for PTC (Fig 3). Similarly, values of DN 60–80 for heterozygous UA phenotype were observed for 100 μM and 30–50 μM of dibucaine for BTC and PTC, respectively. Thus, the optimal differentiation of the serum samples was obtained with 100 μM dibucaine for BTC and 50 μM for PTC.

### Selection of optimal fluoride concentration

**Fig 4:**
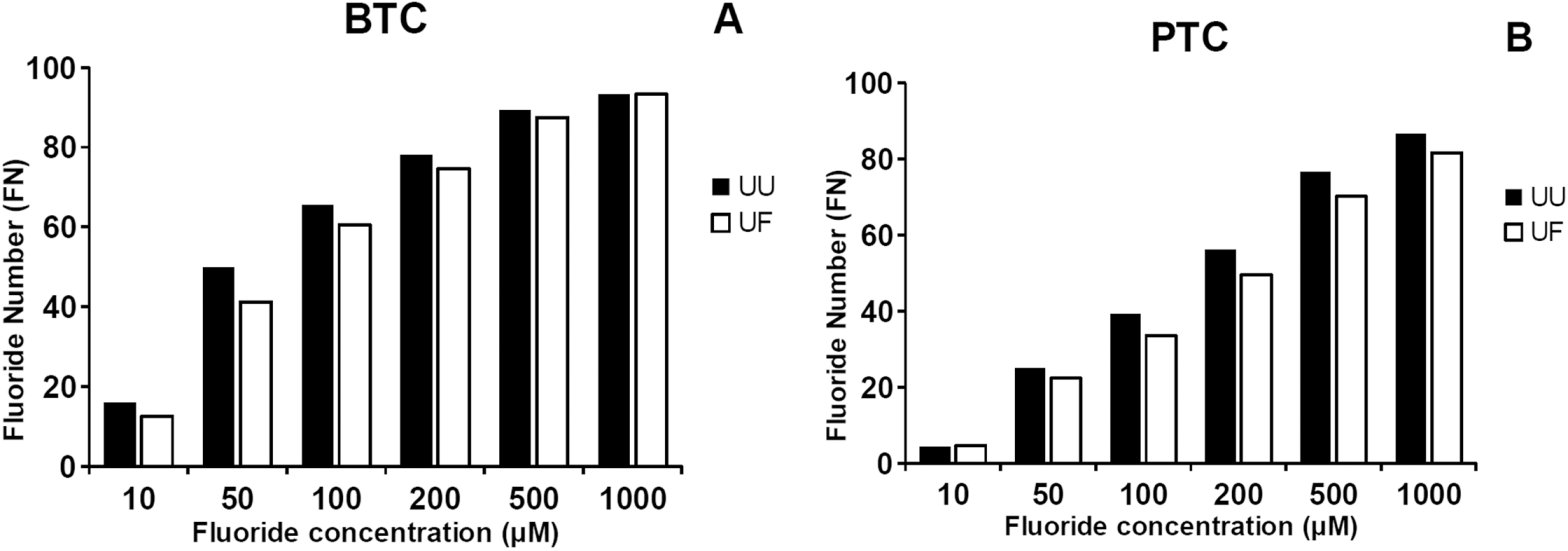
Characteristics of fluoride inhibition of “usual” and “fluoride” forms of BChE. FN values were calculated in assays performed at various dibucaine concentration at 25°C using 5mM BTC (A) or PTC (B) as substrate with two types of 400-fold diluted serum samples from heterozygous individuals with usual UU and UF phenotype. Fluoride Number (FN) = (1 - (butyrylcholinesterase activity in presence of inhibitor/butyrylcholinesterase activity in absence of inhibitor)) x 100. Assays were performed in triplicate, standard error for all points is less than 3% of the values.

To determine the optimal sodium fluoride concentration for measurement of fluoride number (FN), BChE activity was assayed at various sodium fluoride concentrations at 25°C, using 5mM BTC or PTC as a substrate. The assays were performed with two types of 400-fold diluted serum samples: from a patient with usual UU phenotype and from a heterozygous patient with UF phenotype. Values of FN ≥ 50 for UU phenotype are achieved at least at 50 μM concentration of sodium fluoride for BTC and 200 μM sodium fluoride for PTC (Fig 4). The same, expected values of FN less than 50 for heterozygous UF phenotype were observed for 50 μM and 200 μM of sodium fluoride for BTC and PTC respectively. The most appropriate differentiation of fluoride resistant phenotype of BChE in the serum samples was obtained with 50 μM sodium fluoride for BTC and 200 μM for PTC.

## Discussion

The study presents our experience and conclusions improving the routine BChE activity assays. Substrate hydrolysis was monitored by repeated measurements at 1 min intervals for at least 5 minutes by a thermostated microplate reader spectrophotometer at 25°C. The absorbance was measured at 412 nm and a real concentration of TNB ions was evaluated by using Lambert-Beer law with the known molar extinction coefficient of TNB. One unit (U) of enzyme activity is defined as the amount that hydrolyzes one micromole of substrate in 1 minute. The values of BTC hydrolysis rates were corrected for 1 cm path length, dilution factor and extinction coefficient to obtain activity in units per liter of serum, using the equation describing absorbance: A = ε*c*l (also known as Beer-Lambert law), where: **A**–absorbance at 412 nm, **c–**molar concentration [M], **ε** –molar extinction coefficient, **l**–light path length measured in centimeters. The molar extinction coefficient of TNB was originally reported to be 13,600M(-1)cm(-1) at 412nm and pH 8.0. This value has been cited frequently in the literature, but later studies have corrected this value. The molar absorption coefficients of TNB at 412 nm are 14150 M(-1)cm(-1) at 25°C and 13,800 M(-1)cm(-1) at 37°C in 100 mM PB buffer, pH 7.4 [33]. The light path length is fixed at 1 cm by the distance between the walls of the standard cuvette. Using a microplate reader, measurements are taken vertically so the distance the light travels through a sample varies depending on the height of liquid in the plate. Therefore, to calculate TNB concentration using Beer-Lambert law equation, a path length correction value must be used to account for the different light path length corresponding to the sample volume. The path length in the microtiter plates used in the assays was estimated experimentally as 0.58 cm as described in Promega Technical Note [34].

Duration of enzymatic assays should allow to achieve thermal equilibration of the reaction mixture and also to evaluate correctness by monitoring linearity of slopes during the assays. Different spectrophotometers have various limit of signal detection, so the best way is to monitor slopes in real time apparatus. As shown in Fig 1, we recommend to use 400 –fold diluted serum samples since this dilution reached nearly ideal slope for 20 minutes of the assay in 37°C and 25°C for all serum samples presenting low as well as high BChE activity. During our attempts to find a perfect serum dilution, we found the same sample has various BChE activity after calculated corrections in various serum dilutions. Measured enzyme activity is dependent on the factor of serum dilution, the more diluted serum, the higher BChE activity (Fig 2), and this relations were observed in all tested BTC concentrations and also for the PTC (data not shown). Lineweaver–Burk plot of data presented in Fig 2A indicates mixed type of inhibition since Km and Vmax change (Inset Fig 2A), so we tried to figure out these phenomena. Serum samples were dialyzed several times in dialyzing bag or in spin columns at 3kDa cut off, but the effect still persisted (data not shown). This indicates that inhibitor must be larger than 3 kDa, so it could be a protein or other large molecule. It is notable that purified BChE expressed in CHO cells did not show dilution activity dependence, and after dilution and calculated corrections the enzyme had the same activity (data not shown).

During our attempts to measure the rate of BTC hydrolysis at 37°C (15 degrees above ambient temperature), we encountered many problems with temperature control. Assays were performed in temperature controlled plate reader and buffers, tips, plates, pipettes were prewarmed at 37°C prior to assays. Nevertheless, we found observed temperature in wells was about 1–2 degrees lower than set in the reader (37°C). We tried to improve our control over the reaction temperature by performing assays in the thermostated room at 37°C, but still it was not perfect. There are also other factors than we could not control like: temperature gradient in wells, different heat transmission from and to the plate during the assays, different plate thermal geometry (border wells had about 1 degree lower temperature than central wells), heating and cooling parameters of plate reader. More accurate results were obtained when temperature of reaction was lowered to 25°C (3 degrees above ambient temperature). BChE activity at 37°C can be obtained via recalculation by multiplying the activity at 25°C by factor 1.56 [35].

Since there is some confusion about FN and DN number calculations and reference range in literature data [11, 15, 36, 37], we have presented these values for UU, UA, UF phenotype serum samples at different concentrations of these inhibitors. Our results indicate that differentiation of phenotypes can be performed at wide range of inhibitors concentrations. Due to the lack of sharp boundaries between heterozygous UU and UF phenotypes it is sometimes difficult to differentiate a definite phenotype. To obtain accurate results, reference values for each phenotype, inhibitor and its concentration should be established before assays. Type of substrate and concentration of inhibitors have great impact on obtained DN and FN values.

Based on our experience in BChE activity assays and obtained results we postulate that the effect of serum dilution on the enzyme activity should be taken into consideration during approval of an interlaboratory standardized protocol for measuring BChE activity.

## References

[1] Johnson G, Moore SW. Why has butyrylcholinesterase been retained? Structural and functional diversification in a duplicated gene. Neurochem Int 2012;61:783–97. 10.1016/j.neuint.2012.06.016 22750491

[2] Lockridge O. Review of human butyrylcholinesterase structure, function, genetic variants, history of use in the clinic, and potential therapeutic uses. Pharmacol Ther 2015;148:34–46. 10.1016/j.pharmthera.2014.11.011 25448037

[3] Masson P, Lockridge O. Butyrylcholinesterase for protection from organophosphorus poisons; catalytic complexities and hysteretic behavior. Arch Biochem Biophys 2010;494:107 10.1016/j.abb.2009.12.005 20004171 PMC2819560

[4] Jbilo O, Bartels CF, Chatonnet A, Toutant JP, Lockridge O. Tissue distribution of human acetylcholinesterase and butyrylcholinesterase messenger RNA. Toxicon 1994;32:1445–57. 7886701 10.1016/0041-0101(94)90416-2

[5] Manoharan I, Kuznetsova A, Fisk JD, Boopathy R, Lockridge O, Darvesh S. Comparison of cognitive functions between people with silent and wild-type butyrylcholinesterase. J Neural Transm 2007;114:939–45. 17318303 10.1007/s00702-007-0631-x

[6] Brimijoin S, Hammond P. Butyrylcholinesterase in human brain and acetylcholinesterase in human plasma: trace enzymes measured by two-site immunoassay. J Neurochem 1988;51:1227–31. 2901462 10.1111/j.1471-4159.1988.tb03091.x

[7] Eddleston M, Buckley NA, Eyer P, Dawson AH. Management of acute organophosphorus pesticide poisoning. Lancet 2008;371:597–607. 17706760 10.1016/S0140-6736(07)61202-1 PMC2493390

[8] Coye MJ, Lowe JA, Maddy KT. Biological monitoring of agricultural workers exposed to pesticides: I. Cholinesterase activity determinations. J Occup Med 1986;28:619–27. 3746482 10.1097/00043764-198608000-00018

[9] Lockridge O, Masson P. Pesticides and susceptible populations: people with butyrylcholinesterase genetic variants may be at risk. Neurotoxicology. 2000;21:113–26. 10794391

[10] Strelitz J, Engel LS, Keifer MC. Blood acetylcholinesterase and butyrylcholinesterase as biomarkers of cholinesterase depression among pesticide handlers. Occup Environ Med 2014;71:842–7. 10.1136/oemed-2014-102315 25189163 PMC4224972

[11] McGuire MC, Nogueira CP, Bartels CF, Lightstone H, Hajra A, Van der Spek AF, et al Identification of the structural mutation responsible for the dibucaine-resistant (atypical) variant form of human serum cholinesterase. Proc Natl Acad Sci USA 1989;86:953–7. 2915989 10.1073/pnas.86.3.953 PMC286597

[12] Davies RO, Marton AV, Kalow W. The action of normal and atypical cholinesterase of human serum upon a series of esters of choline. Can J Biochem Physiol 1960;38:545–51. 13814417

[13] Nogueira CP, Bartels CF, McGuire MC, Adkins S, Lubrano T, Rubinstein HM, et al Identification of two different point mutations associated with the fluoride-resistant phenotype for human butyrylcholinesterase. Am J Hum Genet 1992;51:821–8. 1415224 PMC1682781

[14] Harris H, Whittaker M. Differential inhibition of human serum cholinesterase with fluoride: recognition of two new phenotypes. Nature 1961;191:496–8. 13711731 10.1038/191496a0

[15] Dietz AA, Rubinstein HM, Lubrano T. Colorimetric determination of serum cholinesterase and its genetic variants by the propionylthiocholine-dithiobis(nitrobenzoic acid)procedure. Clin Chem 1973;19:1309–13. 4758610

[16] Li B, Duysen EG, Lockridge O. The butyrylcholinesterase knockout mouse is obese on a high-fat diet. Chem Biol Interact 2008;175:88–91. 10.1016/j.cbi.2008.03.009 18452903

[17] Lima JK, Leite N, Turek LV, Souza RL, da Silva Timossi L, Osiecki AC, et al 1914G variant of BCHE gene associated with enzyme activity, obesity and triglyceride levels. Gene 2013;532:24–6. 10.1016/j.gene.2013.08.068 24001779

[18] Iwasaki T, Yoneda M, Nakajima A, Terauchi Y. Serum butyrylcholinesterase is strongly associated with adiposity, the serum lipid profile and insulin resistance. Intern Med 2007;46:1633–9. 17917325 10.2169/internalmedicine.46.0049

[19] Valle A, O'Connor DT, Taylor P, Zhu G, Montgomery GW, Slagboom PE, et al Butyrylcholinesterase: association with the metabolic syndrome and identification of 2 gene loci affecting activity. Clin Chem 2006;52:1014–20. 16574762 10.1373/clinchem.2005.065052

[20] Kalman J, Juhasz A, Rakonczay Z, Abraham G, Zana M, Boda K, et al Increased serum butyrylcholinesterase activity in type IIb hyperlipidaemic patients. Life Sci 2004;75:1195–204. 15219807 10.1016/j.lfs.2004.02.019

[21] Alcantara VM, Chautard-Freire-Maia EA, Scartezini M, Cerci MS, Braun-Prado K, Picheth G. Butyrylcholinesterase activity and risk factors for coronary artery disease. Scand J Clin Lab Invest 2002;62:399–404. 12387587 10.1080/00365510260296564

[22] Vaisi-Raygani A, Rahimi Z, Tavilani H, Pourmotabbed T. Butyrylcholinesterase K variant and the APOE-epsilon 4 allele work in synergy to increase the risk of coronary artery disease especially in diabetic patients. Mol Biol Rep 2010;37:2083–91. 10.1007/s11033-009-9666-4 19685167

[23] De Vriese C, Gregoire F, Lema-Kisoka R, Waelbroeck M, Robberecht P, Delporte C. Ghrelin degradation by serum and tissue homogenates: identification of the cleavage sites. Endocrinology 2004;145:4997–5005. 15256494 10.1210/en.2004-0569

[24] Chen VP, Gao Y, Geng L, Parks RJ, Pang YP, Brimijoin S. Plasma butyrylcholinesterase regulates ghrelin to control aggression. Proc Natl Acad Sci USA 2015;112:2251–6. 10.1073/pnas.1421536112 25646463 PMC4343161

[25] Benyamin B, Middelberg RP, Lind PA, Valle AM, Gordon S, Nyholt DR, et al GWAS of butyrylcholinesterase activity identifies four novel loci, independent effects within BCHE and secondary associations with metabolic risk factors. Hum Mol Genet 2011;20:4504–14. 10.1093/hmg/ddr375 21862451 PMC3196893

[26] Das UN. Acetylcholinesterase and butyrylcholinesterase as markers of low-grade systemic inflammation. Ann Hepatol 2012;11:409–11. 22481463

[27] Miao Y, He N, Zhu JJ. History and new developments of assays for cholinesterase activity and inhibition. Chem Rev 2010;110:5216–34. 10.1021/cr900214c 20593857

[28] Ellman GL, Courtney KD, Andres V Jr, Feather-Stone RM. A new and rapid colorimetric determination of acetylcholinesterase activity. Biochem Pharmacol 1961;7:88–95. 13726518 10.1016/0006-2952(61)90145-9

[29] Naik RS, Liu W, Saxena A. Development and validation of a simple assay for the determination of cholinesterase activity in whole blood of laboratory animals. J Appl Toxicol 2013;33:290–300. 10.1002/jat.2730 22407886

[30] Doctor BP, Toker L, Roth E, Silman I. Microtiter assay for acetylcholinesterase. Anal Biochem 1987;166:399–403. 3434781 10.1016/0003-2697(87)90590-2

[31] Worek F, Mast U, Kiderlen D, Diepold C, Eyer P. Improved determination of acetylcholinesterase activity in human whole blood. Clin Chim Acta 1999;288:73–90. 10529460 10.1016/s0009-8981(99)00144-8

[32] Garry PJ. Serum cholinesterase variants: examination of several differential inhibitors, salts, and buffers used to measure enzyme activity. Clin Chem 1971;17:183–91. 5100960

[33] Eyer P, Worek F, Kiderlen D, Sinko G, Stuglin A, Simeon-Rudolf V, et al Molar absorption coefficients for the reduced Ellman reagent: reassessment. Anal Biochem 2003;312:224–7. 12531209 10.1016/s0003-2697(02)00506-7

[34] Calculating Nucleic Acid or Protein Concentration [database on the Internet]. 2009. Available: http://www.promega.com/~/media/files/resources/application notes/pathlength/calculating nucleic acid or protein concentration using the glomax multi microplate instrument.pdf?la=en.

[35] Lothar T. Clinical Laboratory Diagnostics: Use and Assessment of Clinical Laboratory Results, 1st ed. Frankfurt/Main: TH-Books Verlagsgeselschaft, 1998 1727.

[36] Maekawa M, Sudo K, Dey DC, Ishikawa J, Izumi M, Kotani K, et al Genetic mutations of butyrylcholine esterase identified from phenotypic abnormalities in Japan. Clin Chem 1997;43:924–9. 9191541

[37] Kovarik Z, Simeon-Rudolf V. An improvement in segregation of human butyrylcholinesterase phenotypes having the fluoride-resistant variants. Arh Hig Rada Toksikol 2003;54:239–44. 14994645

